# Regulation of DNA Methylation During Plant Endosperm Development

**DOI:** 10.3389/fgene.2022.760690

**Published:** 2022-02-10

**Authors:** Dongdong Lu, Jixian Zhai, Mengli Xi

**Affiliations:** ^1^ Key Laboratory of Forest Genetics and Biotechnology of Ministry of Education, Nanjing Forestry University, Nanjing, China; ^2^ Department of Biology, Southern University of Science and Technology, Shenzhen, China; ^3^ Institute of Plant and Food Science, Southern University of Science and Technology, Shenzhen, China; ^4^ Key Laboratory of Molecular Design for Plant Cell Factory of Guangdong Higher Education Institutes, Southern University of Science and Technology, Shenzhen, China

**Keywords:** DNA methylation, demethylation, imprinted genes, endosperm, symmetrical methylation, non-symmetrical methylation

## Abstract

The endosperm is a vital storage tissue in plant seeds. It provides nutrients to the embryos or the seedlings during seed development and germination. Although the genetic information in the endosperm cannot be passed directly to the next generation, its inherited epigenetic marks affect gene expression and its development and, consequently, embryo and seed growth. DNA methylation is a major form of epigenetic modification that can be investigated to understand the epigenome changes during reproductive development. Therefore, it is of great significance to explore the effects of endosperm DNA methylation on crop yield and traits. In this review, we discuss the changes in DNA methylation and the resulting imprinted gene expression levels during plant endosperm development, as well as their effects on seed development.

## Introduction

In angiosperms, the development of seeds requires double fertilization. The egg and central cells independently combine with sperm cells and develop into the embryo and endosperm, respectively, (Bleckmann et al., 2014). The functions of endosperm are mainly to act as the nutrient supplier, to be the mechanical barrier for the embryo, and to be the growth regulator of the embryo during seed development and germination. The endosperm is also a fundamental factor for the seed prosperity of angiosperms (Yan et al., 2014). Additionally, the endosperm is a critical factor in controlling seed viability and dormancy (De Giorgi et al., 2015). The vast majorities of the edible parts of rice, wheat, and corn, which account for approximately 70% of human food consumption, are endosperm tissues, which are rich in starch, protein, vitamins, dietary fiber, and other nutrients needed in the human diet (Kang et al., 2008). Therefore, improving endosperm contents and quality is a significant issue. Regulation of endosperm development involves gene imprinting and epigenetic modifications. DNA methylation is a major epigenetic modification that participates in gene expression, TE silencing, and genome stability during plant development. It is related to epigenetic transgenerational inheritance. Owing to the importance of the endosperm, its DNA methylation and genome imprinting are reviewed here.

This review introduces the methylation and demethylation of plant endosperm and the imprinted gene expression resulting from DNA methylation. We discuss the effects of endosperm DNA methylation on plant development.

## DNA Methylation and Demethylation of the Plant Endosperm

DNA methylation, the addition of a methyl (CH_3_) group at carbon 5 of cytosine by DNA methyltransferases, is a critical epigenetic marker in mammals and plants (Jin et al., 2011). It represents a heritable change in gene expression not encoded by the DNA sequence. DNA methylation is essential for genomic imprinting, transposable element (TE) silencing, gene regulation, genetic evolution, and genomic stability (Zhang et al., 2018). The loss of DNA methyltransferase function can lead to abnormal plant development (Rajkumar et al., 2020). DNA methylation occurs in three sequence contexts in plants: symmetrical CG and CHG sites and asymmetrical CHH (*H* = C/T/A) sites (Kawakatsu et al., 2017). Different methyltransferases accomplish different DNA methylation patterns through *de novo* methylation and maintenance of methylation (Law and Jacobsen, 2010). There are three types of DNA methyltransferase in plants: DNA Methyltransferase (MET), Domains Rearranged Methyltransferase (DRM), and plant-specific Chromomethylase (CMT). These methyltransferases perform their duties in *de novo* and maintenance methylation, and jointly complete the DNA methylation modification in plants (Ashapkin et al., 2016).

*De novo* methylation refers to the generation of new methylation at sites that have not undergone methylation. The plant-specific RNA-directed DNA methylation (RdDM) pathway catalyzes the *de novo* methylation of three sequence contexts (Matzke and Mosher, 2014). Asymmetric CHH site methylation can only be maintained through *de novo* methylation (Zhang et al., 2018). Some repetitive DNA sequences are transcribed by RNA Polymerase IV (Pol IV) to generate single-strand RNAs (ssRNAs). These ssRNAs produce double-strand RNA (dsRNA) by RNA-Dependent RNA Polymerase 2 (RDR2), which is then cut into 24 nt siRNA by DICER-LIKE 3 (DCL3) (Haag and Pikaard, 2011; Matzke and Mosher, 2014; Zhai et al., 2015). In addition, some inverted repeated DNA sequences can also produce dsRNA under the action Pol II and RDR6, which is further cleaved by DCL3 to produce 21 nt siRNA. These 24 nt and 21 nt siRNAs combine with Argonaute 4 (AGO4) to form a siRNA-AGO4 complex, which recruits DRM1 and DRM2 to *de novo* methylation in the three sites (CG, CHG, CHH) (Matzke and Mosher, 2014). In addition to the RdDM pathway, CMT2 and CMT3 can also catalyze *de novo* methylation (Law and Jacobsen, 2010). The nucleosome remodeling factor Decrease in DNA Methylation 1 (DDM1) changes nucleosome conformation, binds CMT2 to histone H3 lysine 9 dimethylation (H3K9me2) and mediates the *de novo* methylation of the adjacent CHG and CHH sites (Kuo et al., 2017).

Maintenance methylation refers to maintaining the methylation form of the original site in the process of DNA replication. The maintenance of CG methylation in plants is completed by the methylation regulator VIMs (Variation in Methylation, VIM1, VIM2, VIM3) protein and DNA methyltransferase MET1. VIMs recognize and bind to the hemimethylated CG site, recruit MET1 to complete CG methylation of the newly synthesized strand, and finally obtain double-stranded DNA methylation of the CG site (Kawashima and Berger, 2014). The maintenance of CHG methylation is mediated by the CMT3-H3K9me2 pathway. CMT3 binds to two H3K9me2 proteins simultaneously and methylates DNA at nearby CHG sites. The methylated CHG DNA recruits Su (var) Homologue 4 (SUVH4), and the deposition of H3K9me2 markers on the nucleosomes surrounding CHG methylated DNA by SUVH4 creates a CHG–H3K9me2 positive feedback loop (Zhang et al., 2018).

In addition, there is an active DNA demethylation process in flowering plants, and this is achieved by DNA glycosylase/lyase through a base excision repair (BER) mechanism. Three types of DNA glycosylases have been found in plants: Demeter (DME), Repressor of silencing1 (ROS1), Demeter-like (DML2 and DML3). Thus, the final methylation level in the genome is determined by the activities of both DNA methyltransferases and demethylases (Gong et al., 2002).

**FIGURE 1:**
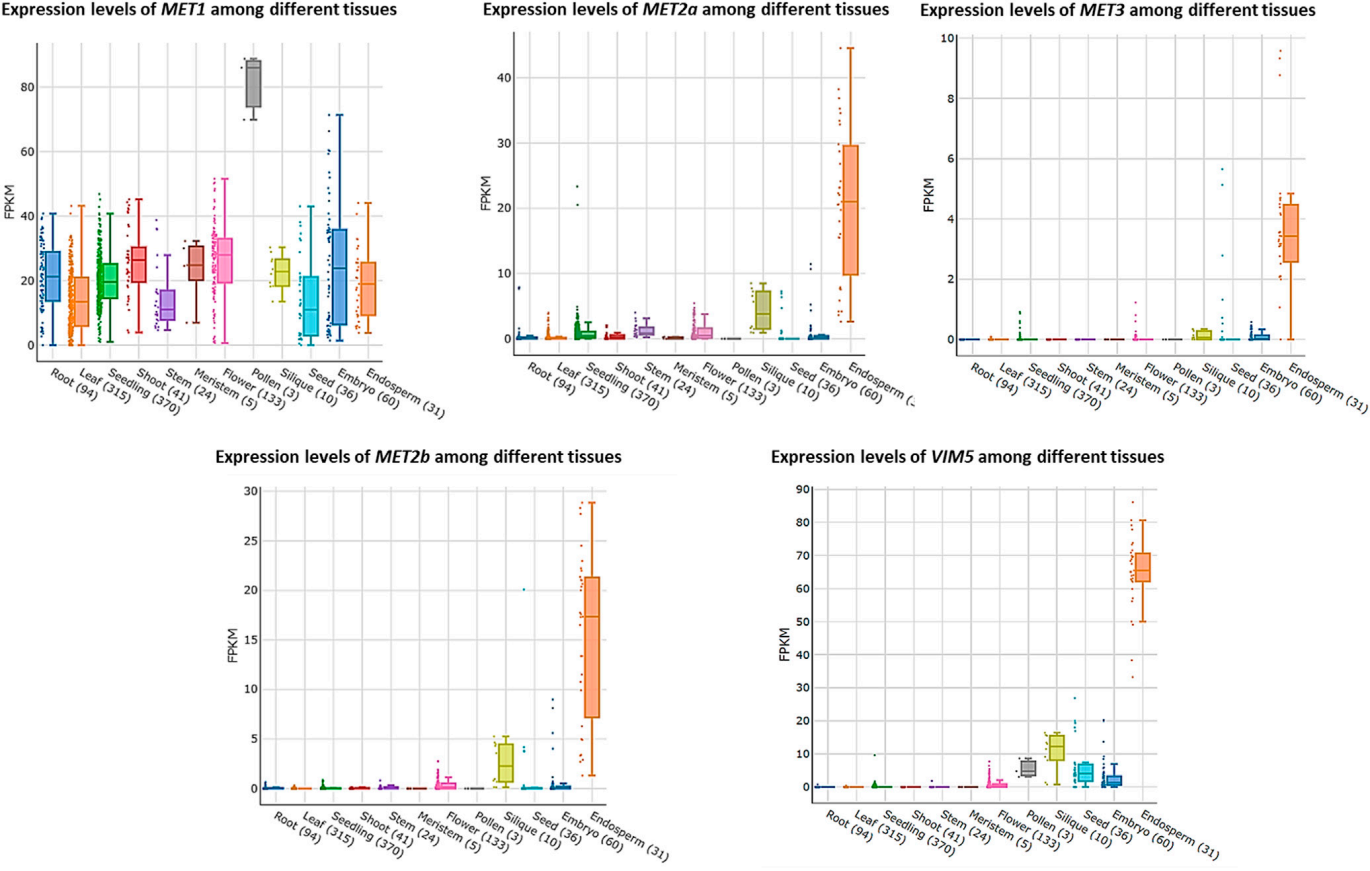
Expression levels of *MET1*, *MET2a*, *MET2b*, *MET3* and *VIM5* among different tissues.

**FIGURE 2:**
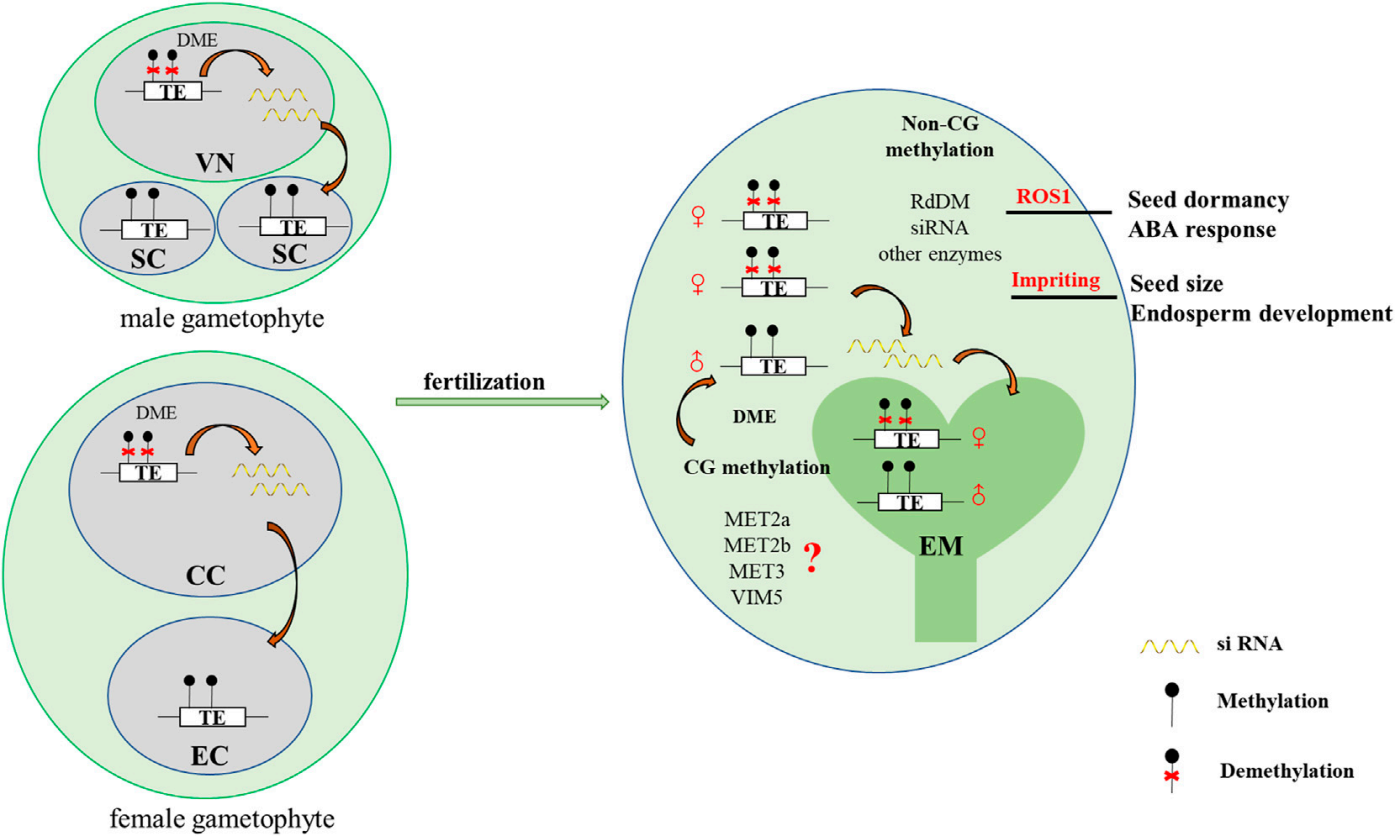
A simplified model of DME-mediated DNA demethylation in *Arabidopsis* endosperm. Before fertilization, the central cell and vegetative cell are highly demethylated resulted from the action of DME. DME preferentially targets TE regions (Gehring et al., 2009; Hsieh et al., 2009; Ibarra et al., 2012). The vegetative cell produces siRNA into the sperm cells and maintains the sperm cell hypermethylation through the RDdM pathway (Martinez et al., 2016). So the methylation level in the endosperm is much lower than in the embryo after fertilization. The siRNAs produced by the demethylation of the endosperm are transferred to the embryo to maintain the stability of the embryo genome. MET2a, MET2b, MET3, and VIM5 may jointly regulate CG methylation in the endosperm. In addition, the differential methylation of the embryo and endosperm leads to imprinting in the endosperm, which may affect endosperm development and control seed size. Additionally, the endosperm demethylase ROS1 regulates seed dormancy. VN: vegetative cell nucleus, SC: sperm cell, CC: central cell, EC: egg cell, EM: embryo.

In *Arabidopsis*, the expression levels of the major DNA methylation enzymes are available at the *Arabidopsis RNA-seq database* (http://ipf.sustech.edu.cn/pub/athrna/). In wild-type endosperm, the expression of *MET1* is low, whereas the expression levels of *MET2a* and *MET2b*, which are specifically expressed in central cells, are high. The paternal imprinting genes *VIM5* and *MET3* are also specifically expressed and highly expressed in the endosperm. Therefore, we speculate that *MET2a*, *MET2b*, *MET3*, and *VIM5* may jointly regulate CG methylation in the endosperm (Figure 1), which requires further experimental proof. We profile a simple model based on DME-mediated DNA demethylation in the endosperm (Figure 2). The *DME* gene is predominantly expressed in the central cell, and DME induces global hypomethylation (Choi et al., 2002; Hsieh et al., 2009). Before fertilization, the central cell and vegetative cell are highly demethylated resulted from the action of DME. DME preferentially targets TE regions (Hsieh et al., 2009; Ibarra et al., 2012). The demethylation of the maternal genome during gametogenesis is also reported in other species—castor bean (Park et al., 2016), rice (Zemach et al., 2010; Park et al., 2016), and maize (Lauria et al., 2004). The vegetative cell produces siRNA into the sperm cells and maintains the sperm cell hypermethylation through the RDdM pathway (Martinez et al., 2016). So the methylation level in the endosperm is much lower than in the embryo after fertilization. The siRNAs produced by the demethylation of the endosperm are transferred to the embryo to maintain the stability of the embryo genome (McCue et al., 2012). The loss of *DME* function (*dme* mutant) in endosperm restores CG methylation but unexpectedly further diminishes non-CG methylation, suggesting demethylation in a non-CG context is regulated by a yet unknown DME-independent mechanism (Hsieh et al., 2009; Jullien et al., 2012).

## The Level of DNA Methylation in Endosperm Varies Among Different Plant Species and During Their Development

**FIGURE 3:**
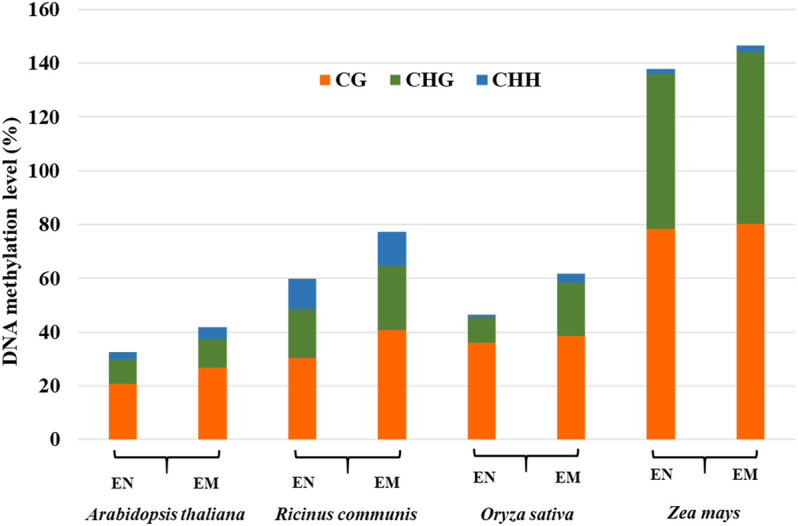
DNA methylation levels of CG, CHG, and CHH in the endosperm (EN) and embryo (EM) among different species.

The endosperm of some plants, such as *Arabidopsis*, only exist in the early stage of seed development and gradually disappear with seed development (Brown et al., 1999). Most monocotyledons, some dicotyledons, and gymnosperms, have endosperm in their mature seeds, such as castor beans and rice (Greenwood and Bewley, 1982; Brown et al., 1996). The DNA methylation profiles in the endosperm of different plants are significantly different, suggesting that DNA methylation profiles of endosperm are not conserved. By comparing the methylation levels of the endosperm among different plants, it was found that genomic DNA hypomethylation in endosperm relative to the embryo is widespread (Figure 3), especially in dicotyledons (Hsieh et al., 2009; Zemach et al., 2010; Lu et al., 2015; Xu et al., 2016). CG, CHG, and CHH methylation levels were low at 4 days after pollination (DAP), but all three contexts of DNA methylation levels were elevated at 6 days after pollination by DNA methylation sequencing in *Arabidopsis* endosperm (Pignatta et al., 2014; Moreno-Romero et al., 2016). Hu et al. found that the methylation levels were higher during the early (3–5 DAP) and late stages (13–25 DAP) of endosperm development compared with the middle stage (7–11 DAP) in maize endosperm (Hu et al., 2021). Thus, DNA methylation represents a dynamic process during endosperm development. The DNA methylation changes in the endosperm affect the expression of genes and siRNAs, thereby affecting endosperm formation and seed development (Moore et al., 2013).

## Genomic Imprinting by DNA Methylation During Plant Endosperm Development

Genomic imprinting is the process of inheriting the epigenetic marking for a particular segment of a chromosome from paternal or maternal alleles (Feil and Berger, 2007). The endosperm is the main organ that undergoes genomic imprinting in flowering plants (Gehring et al., 2011; Hsieh et al., 2011). The epigenetic regulation of genomic imprinting plays an indispensable role in normal endosperm development and seed fertility. The misregulation of imprinted genes affects the sizes of seeds or leads to inviable seeds (Tiwari et al., 2010; Hornslien et al., 2019). The generation of genomic imprinting is mainly caused by the different epigenetic modifications of male and female gametes before fertilization (Batista and Köhler, 2020). When the central cell and sperm cell fuses to form the primordial endosperm nucleus, the differences in epigenetic modification between the male and female genomes result in only one allele being expressed and the other being silenced. The differential loss of DNA methylation in the paternal and maternal alleles produces different chromatin marks in *Arabidopsis*. For example, the methylated paternal allele can lead to being transcriptionally silent, while the demethylation of maternal allele would become a transcriptionally active state (Kinoshita et al., 2004; Jullien et al., 2006; Tiwari et al., 2008). In *Arabidopsis*, the expression level of the DNA methyltransferase gene *MET1* is low in central cells, whereas the expression level of the demethylase gene *DME* is high (Huh et al., 2008). Therefore, the central cells maintain a lower DNA methylation level, but the sperm cells maintain a higher DNA methylation level because *DME* is not expressed (Huh et al., 2008).

RdDM is also critical for silencing of the paternal allele at MEG (maternally expressed imprinted genes) loci; Vu et al. used Col and Cvi to distinguish the parental alleles of *Suppressor OF drm1 drm2 cmt3 (SDC)* and *MOP9.5* (also called *AtPI4Kγ3*, a type II phosphoinositide 4-kinase), and crossed wild-type ovules with pollen from mutants for RdDM (such as *nrpd2a* mutant, NRPD2A is the second largest subunit of RNA pol IV and pol V); they observed activation of *SDC* and *MOP9.5* paternal alleles from *nrpd2a* homozygous plants. Further research found that maternal-specific expression of imprinted genes *SDC* and *MOP9.5* was maintained by MET1. These results suggest that small RNAs have a significant role in setting MEG expression patterns (Vu et al., 2013). PEGs (paternally expressed imprinted genes) can also be hypomethylated at the maternal allele and hypermethylated at the paternal allele (Hsieh et al., 2009; Zhang et al., 2014). So the maternal hypomethylation is essential for the silencing of the maternal allele for many PEGs (Hsieh et al., 2011; Wolff et al., 2011). MEGs are generally more affected by DNA methylation than PEGs (Chen et al., 2018), but the latter is also regulated by histone modification, such as H3K27me3 (Wolff et al., 2011; Zhang et al., 2014). Two other repressive epigenetic marks, H3K9me2 and CHG methylation, also contribute to maternal alleles silencing of PEGs, leading to differential expression of parent-of-origin alleles in the endosperm (Inoue et al., 2017; Moreno-Romero et al., 2019). Silencing of the maternal *PHERES 1* (*PHE1*, a paternally expressed imprinted transcription factor gene) allele depends on the Polycomb Repressive Complex 2 (PRC2), and maternally inherited mutations that encode PRC2 proteins cause biallelic expression of *PHE1* (Kohler et al., 2005). The differences in the expression of PRC2 between sperm and central cells resulted in different histone methylation modifications of parental genomes in the endosperm (Luo et al., 2000; Schoft et al., 2011). DNA methylation can prevent H3K27me3 modification and interfere with PRC2 function (Weinhofer et al., 2010; Deleris et al., 2012; Jermann et al., 2014).

Genomic imprinting disruption accompanies endosperm abortion, and the expression of many imprinted genes also changes (Jullien and Berger, 2010; Kradolfer et al., 2013; Florez-Rueda et al., 2016; Tonosaki et al., 2018). Many MEGs affect seed development by regulating endosperm cytogenesis (Niu et al., 2020; Cheng et al., 2021; Tonosaki et al., 2021); whereas most PEGs knock-out mutations generally do not affect normal plant growth and development in *Arabidopsis*. But PEGs are important for endosperm development in plants, several *peg* mutants: such as *adm* (*ADMETOS*) and *peg2* (*At1g49290*) mutants—can rescue triploid seed abortion (Wolff et al., 2015). And the loss of some PEGs can also lead to serious phenotypic defects. For example, the mutants of *PEG1* (*Os01g08570*, encoding an oxygenase dependent on ketoglutarate and iron), *PEG2* (*OsFBX365*, encoding an F-box domain protein), and *PEG3* (*OsFBDUF48*, encoding a DUF295-domain protein) in rice can reduce starch content and seed fertility (Yuan et al., 2017). The PEGs may be directly involved in regulating reproductive isolation between species. In the endosperm of distant *Arabidopsis* inter-accession crosses (such as Columbia × Nossen), the expression disorder of PEGs is more significant than that of MEGs (Wolff et al., 2015). In interploidy crosses, some PEGs mutants rescue seed abortion, so they have a dramatically different phenotype than WT (Kradolfer et al., 2013; Wang et al., 2018). Hundreds of possible imprinted genes have been discovered in plants. However, there is still a lack of in-depth research on the biological functions of plant imprinted genes, even though many imprinting genes co-localize with yield-related traits (Yuan et al., 2017). For example, Chen et al. found that the rice grain weight QTL—*Grain Weight 2*—mainly expressed maternal alleles in the endosperm (Chen et al., 2016; Niu et al., 2020). These studies indicate that both MEGs and PEGs can participate in plant endosperm development.

## Discussion

The DNA methylation of endosperm plays a vital role in regulating seed development and storage material biosynthesis. The removal of imprinted genes can affect endosperm development and lead to seed abortion. In addition, DNA methylation can also regulate endosperm development by regulating the expression of genes and small RNAs. For example, DNA methylation affects starch synthesis in maize endosperm (Hu et al., 2021). DNA methylation also regulates seed size (Rajkumar et al., 2020) and dormancy (Zhu et al., 2018), and it directly affects crop yield and quality. At present, the research on most crops is limited to the regulation of transcription factors, and the research on DNA methylation mainly focuses on model organisms. Although the methylation sequencing of plant endosperm is gradually increasing, the regulatory pathways related to DNA methylation and demethylation in the endosperm are unclear. Therefore, it is recommended to use a combination of methylation sequencing and RNA sequencing (RNA sequencing, single-cell sequencing, small RNA sequencing) to study plant endosperm and establish a complete regulatory network profile. It is of great value to identify the cellular heterogeneity of methylation in plants, but it is still extremely challenging to sequence single-cell DNA methylation in plant endosperm. On the one hand, the presence of seed coat makes it difficult to separate pollution-free endosperm. On the other hand, it is difficult to use bisulfite-transformed DNA fragments by library construction and sequencing for highly methylated and highly repetitive genomes. The regulation of methylation in plant endosperm should be the focus of future research.
